# A Bill to Regulate the Practice of Medicine in Maryland

**Published:** 1890-03

**Authors:** 


ARTICLE IV.

### A BILL TO REGULATE THE PRACTICE OF MEDICINE IN MARYLAND.

The following medical bill is now a law regulating the practice of medicine in the State of Maryland:

A bill entitled an Act to repeal sections 39, 40, 41, 42, 43, 44, 45, 46 and 47, of Article 43, of the Code of Public General Laws, title "Health," sub title "Practitioners of Medicine," and to re-enact the same with amendments; and to add additional sections to said Article, under said sub-title, to come in after section 47, and to be designated as sections 48 and 49.

SECTION 1. Be it enacted by the General Assembly of Maryland, that sections 39, 40, 41, 42, 43, 44, 45, 46 and 47, of Articles 43, of the Code of Public General Laws, title "Health," sub-title "Practitioners of Medicine," be and the same are hereby repealed and re-enacted with amendments, so as to read as follows:

SEC. 39. And be it further enacted, That every person, not now practicing medicine, who shall hereafter begin to practice medicine in any of its departments, except dentistry, in the State of Maryland, shall possess the qualifications required by this Act.

SEC. 40. And be it further enacted, That a State Board of Medical Examiners consisting of twelve members, be appointed, composed of two sections, known as the "Regular and Homœopathic Sections of the Medical Examiners Board," each section to have exclusive right to examine and pass upon the qualifications of its own applicants, said members of the regular section to be appointed by the Medical and Chirurgical Faculty of Maryland, of which two shall be from the counties of the Eastern Shore, and five from the Western Shore, of which latter number two shall be from the counties west of the Blue Ridge

Mountains; and said Homœopathic section to be composed of five physicians, appointed by the Maryland State Homœopathic Medical Society, of which three shall be residents of Baltimore, and two of the State at large; the appointees shall be physicians actually engaged in the practice of medicine, and of recognized ability and honor; the term of office shall be four years, or until their successors are appointed and qualified; the term of office of the Board first appointed shall commence on the first Tuesday in May, 1890; no member of any college or university, and no physician having a pecuniary interest in the trade of pharmacy shall be appointed to serve as a member of said Board; vacancies occuring in such for unexpired terms shall be filled by the Board, in accordance with the foregoing provisions of this section; and for expired terms in same manner as for first appointees.

Sec. 41. And be it further enacted, That the Board of Medical Examiners shall meet within thirty days after receiving official notice of their appointment, and proceed to organize by electing a President, Secretary and Treasurer. It shall have a seal, and the Secretary shall be empowered to administer oaths in taking testimony upon any matter pertaining to the duties of said Board. Said board shall hold an annual meeting in the City of Baltimore on the fourth Tuesday in May, and at such other time and places as the President of the Board may deem expedient. Eight members shall constitute a quorum. The board shall keep an official register of all applicants for examination for a license to practice medicine and surgery in this State, said register for license shall show the name, age and last place of residence of each candidate, the school from which he or she may have graduated, and whether such applicant was rejected or licensed under this Act, but such matters shall not be written in said register or made public until after the examination.

Sec. 42. And be it further enacted, That it shall be the duty of said board of Medical Examiners to prepare a schedule of written examinations upon anatomy, Physiology, Chemistry, Surgery, Practice of Medicine, Materia Medica, and Therapeutics, Obstetrics, Gynæcology, Pathology, Medical Jurisprudence and Hygiene. All persons commencing the practice of medicine or surgery in any of its branches after the passage of this Act by the General Assembly shall apply to said Board of Medical Examiners for a license so to do, and when said applicant shall have passed an examination as to proficiency satisfactory to said Board, the President shall grant to such applicant a license to practice medicine and surgery in the state of Maryland.

SEC. 43. And be it further enacted, That all examinations shall be conducted in such manner that the name, school of graduation and preparatory training of said applicant shall not be made known to the Board of Examiners until his examination papers have been graded in accordance with the standard and rules arranged by said Board. The examination shall be fundamental in character and such as can be answered in common by all schools of practice. The votes of all examiners present shall be "yes" or "no," written with their signatures upon the backs of the examination papers of each candidate for the respective branches. An applicant receiving a majority of the votes of the section of said Board before whom the applicant appears, shall be considered to have passed a satisfactory examination and entitled to the license of the said Board.

SEC. 44. And be it further enacted, That a fee of ten dollars shall be paid to the Treasurer of said Board by each applicant before such examination is had which said fee shall be applied toward paying the expenses of the Board.

SEC. 45. And be it further enacted, That the Board may by two thirds vote refuse to grant or may revoke a license for the following named causes, to wit: chronic and persistent inebriety, the practice of criminal abortion, or for publicly advertising special ability to treat or cure diseases which, in the opinion of said Board it is impossible to cure. All parties charged with such offences shall be given a hearing before said Board in person or by attorney, and can appeal from the decision of said Board in person.

SEC. 46. And be it further enacted, That the Board shall refuse to grant a license to any applicant who may be radically deficient in his examination in any essential branch.

SEC. 47. And be it further enacted, That any person receiving a license from said Board shall file the same, or a certified copy therefore with the clerk of the Circuit Court of the county or city in which he or she may reside, and it shall be the duty of said Clerk to register the name of such person, and the president of the board signing the same in a book kept for the purpose, as a part of the records of his office; the fee for each registration shall be one dollar, to be paid by the person whose name is registered.

SEC. 48. And be it further enacted, That this act shall not apply to commissioned Surgeons of the United States Army, Navy or Marine Hospital service, to physicians or surgeons in actual consultation from other States, or to persons temporarily practioing under the supervision of an actual medical preceptor.

SEC. 49. And be it further enacted, That any person to whom the provisions of this act applies, practising or attempting to practice medicine or surgery in this State, without first having obtained the license of said Board of Medical Examiners, shall be guilty of a misdemeanor, and shall pay a fine of not less than fifty dollars, nor more than two hundred dollars for each offense, or in default of payment shall be confined in the county jail until the fines and and costs are paid, and shall be debarred from recovering compensation for services rendered as such physician or surgeon.

SEC. 50. And be it enacted, That all Acts, or parts of Acts now existing, not in accordance with the provisions of this Act are hereby repealed.

SEC. —. And be it further enacted, That the provisions of this Act shall not apply to any midwife or person who may render gratuitous services in cases of emergency.

SEC. —. And be it further enacted, That it is provided that said Board shall make a written report to the Medical and Chirurgical Faculty of Maryland and to the President of the Homœopathic State Medical Society every two years.

SEC. —. And be it further enacted, That this Act shall take effect from the date of its passage.

